# Users’ Perspectives on a Picture Archiving and Communication System (PACS): An In-Depth Study in a Teaching Hospital in Kuwait

**DOI:** 10.2196/medinform.5703

**Published:** 2016-06-15

**Authors:** Ali Jassem Buabbas, Dawood Ameer Al-Shamali, Prem Sharma, Salwa Haidar, Hamza Al-Shawaf

**Affiliations:** ^1^Faculty of MedicineCommunity Medicine and Behavioral SciencesKuwait UniversityHawallyKuwait; ^2^Mubarak AlKabeer HospitalDiagnostic RadiologyMinistry of HealthHawallyKuwait; ^3^Health Sciences CenterVice President OfficeKuwait UniversityHawallyKuwait; ^4^Allied Health SciencesHealth Information AdministrationKuwait UniversityHawallyKuwait

**Keywords:** PACS evaluation, user perspective, IS success, imaging informatics, radiology

## Abstract

**Background:**

Picture archiving and communication system (PACS) is a well-known imaging informatics application in health care organizations, specifically designed for the radiology department. Health care providers have exhibited willingness toward evaluating PACS in hospitals to ascertain the critical success and failure of the technology, considering that evaluation is a basic requirement.

**Objective:**

This study aimed at evaluating the success of a PACS in a regional teaching hospital of Kuwait, from users’ perspectives, using information systems success criteria.

**Methods:**

An in-depth study was conducted by using quantitative and qualitative methods. This mixed-method study was based on: (1) questionnaires, distributed to all radiologists and technologists and (2) interviews, conducted with PACS administrators.

**Results:**

In all, 60 questionnaires were received from the respondents. These included 39 radiologists (75% response rate) and 21 technologists (62% response rate), with the results showing almost three-quarters (74%, 44 of 59) of the respondents rating PACS positively and as user friendly. This study’s findings revealed that the demographic data, including computer experience, was an insignificant factor, having no influence on the users’ responses. The findings were further substantiated by the administrators’ interview responses, which supported the benefits of PACS, indicating the need for developing a unified policy aimed at streamlining and improving the departmental workflow.

**Conclusions:**

The PACS had a positive and productive impact on the radiologists’ and technologists’ work performance. They were endeavoring to resolve current problems while keeping abreast of advances in PACS technology, including teleradiology and mobile image viewer, which is steadily increasing in usage in the Kuwaiti health system.

## Introduction

Picture archiving and communication system (PACS) is a well-known imaging informatics application in health care organizations, specifically designed for the radiology department. A PACS could be defined as “an electronic information system (IS) used to acquire, store, transmit, and display medical images” [1]. Using PACS in hospitals has innumerable benefits at various levels [2]. At the management level, this technology has direct implications for cost reduction, rendering the film production process redundant. At the departmental level, the technology enhances productivity, as all tasks are performed digitally and swiftly; at the clinical level, image interpretation and diagnosis become more precise and accurate [3]. For these reasons, health care organizations are increasingly adopting PACS in their clinical radiology departments, despite the high costs, to benefit from the full advantages of using the technology. PACSs are currently being applied in many medical imaging projects around the world, such as in the United States, the United Kingdom, and Asia. However, the available literature reveals gaps with regard to the systems’ effectiveness and efficiency concerning their intended use.

The existing literature is abounding with studies evaluating PACS [4]. However, these evaluations invariably had different focus and objectives; for instance, there are studies on PACS before and after the system’s implementation [5], users’ satisfaction [6], PACS acceptance [7], cost-effectiveness [8,9], and the system’s efficiency concerning its use and in saving time [10]. The most widely used form of PACS evaluation concerns its impact on users [4,11,12].

In PACS research and practice, once the system has been adopted and implemented, it becomes imperative to evaluate the technology’s effectiveness within an organization [13]. For all practical purposes, evaluation could be defined as “the process of describing the implementation of an information resource and judging its merits and worth” [14]. IS deployment may invariably lead to unintended consequences, affecting the chances of the technology’s success [14]. Several researchers have, therefore, recommended evaluation studies specially focused on PACSs to assess its impact in clinical practice [4,15].

It is of paramount significance to investigate the success of PACS, exploring the factors responsible for the success or failure to determine its worth clinically, based on the direct users of this system.

The conceptual basis of this study is focused on this: the impact of PACS was assessed in a regional hospital in Kuwait based on specific criteria. The study is the first of its kind in Kuwait, there being a scarcity of literature in this field.

### Research Questions

The research questions were specifically as the following: (1) What impact does the PACS have on the clinical practice of radiologists and technologists in the radiology department of Mubarak Al-Kabeer Hospital? (2) Has the use of the PACS proven successful in improving the radiology department’s work performance?

This study aimed at evaluating the success of the PACS in clinical practice, in a bid to determine the technology’s merits for radiologists and technologists, including its drawbacks.

## Methods

### Research Setting

The universe of this study was Mubarak Al-Kabeer Teaching Hospital, which is 1 of the 5 regional hospitals in the State of Kuwait. Table 1 presents the site’s profile. This general hospital is a University-teaching hospital in Kuwait and was chosen because it is always at the forefront of development and advanced medicine. Therefore, to ensure the full advantage of the health information system (HIS), the PACS’s success needed to be verified. The PACS was first introduced in the radiology department of Mubarak Al-Kabeer Hospital in 2004, marking the transition of clinical services from a film-based system, to an electronic-based system. The PACS used is an off-the-shelf, Oracle-based HIS (GE Centrisity RIS i 4.2 plus, GE PACS IW 3.7.3.9 SP 3). The PACS currently has 35 workstations, with a server capacity of 64 terabytes. Radiologists use the PACS to view images through the radiology information system (RIS), which they use to report their cases. The reports generated by the RIS are then sent to the PACS, through which final reports can be sent to HIS. The treating physician needs to submit an access request to see patients’ images on the PACS. In June 2013, the PACS software was upgraded, and currently the system is fully integrated technically with the RIS and the HIS, providing the users with a secured system.

**Table 1:** Mubarak Al-Kabeer teaching hospital’s profile.

Categories	No.
Hospital beds	734
Hospitalized patients	21,124
Physicians	559
Radiologists	52
Radiology technologists	34
PACS administrators	5
Average no. of images examined monthly	32,787

### Study Design

An in-depth study was conducted by using quantitative and qualitative methods. This mixed-method study was based on: (1) survey questionnaires, which were distributed to gather information from radiologists and technologists in the radiology department of Mubarak Al-Kabeer Hospital and (2) semi structured interviews, which were conducted to gather empirical information from the PACS administrators. Ethical approval for the study was obtained from the research department of the Ministry of Health, Kuwait.

To gather the responses of radiologists and technologists concerning the use of the PACS in their clinical practice, a validated questionnaire from a previous study was used [16]. The questionnaire was translated from French into English through an official translation office in Kuwait. The English version of the questionnaire was pretested with 5 radiologists and 3 technologists to ensure the suitability and usability of the questions. Accordingly, a number of amendments were made to the questionnaire. These included excluding questions that were found to be irrelevant to the technologists’ use of the PACS, which comprised items that focused on retrieving, displaying, comparing, and manipulating of images, including confidence level. In addition, a 7-point Likert scale was changed to 5 points to make it easier and more familiar for the respondents.

**Figure 1:**
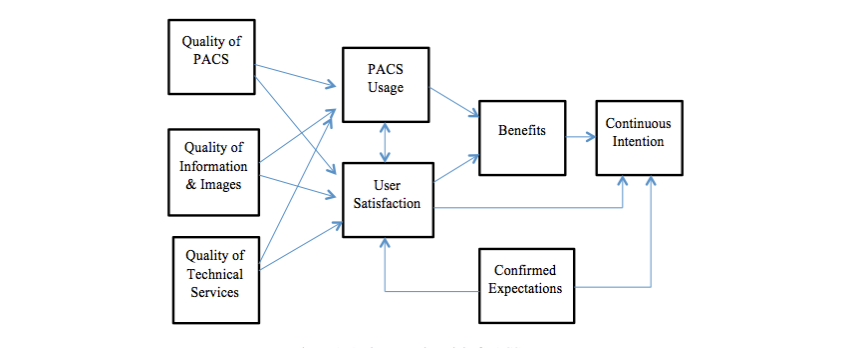
An integrated model of picture archiving and communication system (PACS) success.

In this study, evaluating the PACS’s success was based on an integrated multidimensional model, which was constructed from the model primarily developed by Delone and Mclean [17,18], and later it was developed in which 2 constructs were added to the model, namely: system continuance intention and confirmation of expectations [16] (Figure 1).

The questionnaire comprised 7 sections (Textbox 1) for assessing the users’ perspectives on 8 interrelated dimensions of the PACS success model. These included: (1) perceived system quality; (2) perceived information quality; (3) perceived service quality; (4) system usage; (5) user satisfaction; (6) perceived net benefits; (7) system continuance intention; and (8) confirmed expectations. The questionnaire was distributed to all radiologists and radiology technologists who had used the PACS in their clinical practice for the last 2 years.

**Sections of the questionnaire.**

Section 1: Quality of PACS

Ease of access and use

- Diversity of functionalities offered by the PACS
- Reliability of the hardware and software
- PACS integration and compatibility with the RIS and the HIS
- Security of the PACS

The data gathered through the questionnaire were complemented by conducting semi structured interviews with PACS administrators to gain an understanding of the prevailing clinical environment, which entails them communicating with radiologists, doctors, and technologists, including providing information technology services and support [19]. Their experience further enriched the information gathered and the study’s purpose.

The interviews’ focus was primarily similar to that of the questionnaire: to gain a deeper insight into the response patterns of the respondents. The interviews were conducted with the radiology technologists, who are responsible for administering the PACS and overseeing the RIS operations in the radiology department.

### Statistical Analysis

Data management, analysis, and graphical presentation were carried out using the software Statistical Package for the Social Sciences (SPSS), version 22.0. The questionnaire was evaluated for internal consistency and reliability, and Cronbach alpha values were estimated for major perspectives by combining the Likert scale items for specific aspects, including quality, information, images, technical support and usage, user satisfaction, and overall opinion on the PACS. The descriptive statistics analysis generated frequencies and percentages for all the 5-point Likert scale items (1 as lowest or strongly disagree and 5 as highest or strongly agree) in the questionnaire. The Likert scale data were also analyzed to find average values for overall responses and to compare the mean (±standard deviation, SD) between radiologists and technologists using *t* tests or nonparametric Mann-Whitney tests. The quantitative or continuous variables, age, duration of use (h), and minutes saved every day were first ascertained for normal distribution, applying the Kolmogorov-Smirnov test and were presented as mean ± SD and range for normally distributed variables and as median, range and interquartile (IQ) for skewed data. The chi-square or Fisher exact test was applied to find any association or significant difference between categorical variables. The Spearman correlation coefficient (rho) was used to find any correlations among the number of hours worked, the use of the PACS, and the minutes saved in daily practice. The 2-tailed probability value *P*<.05 was considered statistically significant.

## Results

### Questionnaires

#### Respondent Demographics

The study’s overall response rate was 70%: 75% of the radiologists and 62% of the technologists of the radiology department. The study had 60 respondents: 39 radiologists (mean age = 36±7.5 SD) and 21 technologists (mean age = 28±10 SD). The respondents’ ages varied between 20 and 60 years, with the majority (85%; 51 of 60) aged younger than 40 years. The respondents’ average self-rated level of familiarity with computers was 4.8 ± 1.34 (mean ± SD) on a scale of 1-7, and 41% (24 of 59) of the respondents had earlier experience with PACSs before working at this radiology department.

#### Evaluation of Different Perspectives on the PACS

The overall responses on different perspectives were analyzed, and composite reliability and coefficients (Cronbach alpha) were computed and presented in Table 2, along with mean and range for each perspective. The Cronbach alpha values ranged between.73 and.96, except for one as shown in in Table 2. System quality, images produced, and services, all had high (>.9) Cronbach alpha values.

The overall perspectives of users have been presented on the following aspects:

##### System Quality

Almost three-quarters (75%; 44 of 59) of the respondents rated the PACS positively and as user friendly, with a mean of 3.28 (Table 2). Comparatively fewer (64%; 38 of 59) respondents mentioned some drawbacks of the system, such as it being temporarily out of service or not working, numerous bugs, waiting time at the workstations, and the screen quality slowing PACS use. The majority (81%; 48 of 59) agreed that the PACS had improved the quality of services at the radiology department (mean=4.01). However, some suggestions were provided by respondents (mean=3.57) with regard to the system’s improvement included the provision of more options and investment in upgrading the visualization equipment (PC monitors).

##### Information Quality

In all, 90% (53 of 59) agreed that the PACS produced better and higher-quality information (mean=3.75) that was accurate, updated, relevant, and timely. The system also provided complete patient information, including adequate access to patients’ historical data (mean=3.56).

##### Image Quality

The PACS users were extremely satisfied with regard to the quality of the images produced, ease of understanding, and relevance (mean=4.27). They found that the PACS produced much better images compared with traditional films (mean=4.33).

##### Technical Support and Services

The PACS users were quite satisfied with technical support (mean=3.60) and the reliability, promptness, and dependability of services.

##### Use of PACS and Satisfaction

**Figure 2:**
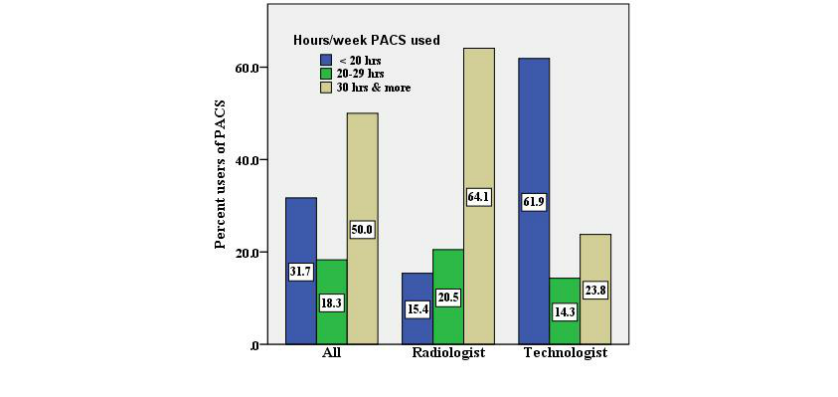
Respondents’ picture archiving and communication system (PACS) use per week.

In all, 50% (30 of 60) of the respondents mentioned using the PACS for more than 30 hours per week (Figure 2), although a significant difference was found regarding the duration of PACS use (hours/week) between radiologists and technologists (*P*<.001). A high level of user satisfaction was shown with regard to their experience in using the PACS (mean=3.65). The usage of various tools, including making changes to the display format, retrieving and “split screen” to compare images was found to be quite satisfactory (mean=3.57), especially among radiologists.

##### Future Use and Expectations on PACS

In all, 83.9% (mean=3.39) of PACS users mentioned their expectations better than what they expected originally and showed intention to continue using PACS.

##### Overall Opinions and Impact of PACS

Based on 21 different statements, 93% (56 of 60) of the PACS users showed consensus on various aspects of the system’s benefits and effectiveness (mean=4.01), and the mean was significantly higher for technologists as compared with radiologists (4.22 vs 3.89). Furthermore, the results showed that 80% (48 of 60) of the PACS users reported saving more than 30 minutes of their practice time each day, whereas 38% (23 of 60) mentioned saving more than an hour each day.

**Table 2:** PACS users and their responses.

User perspectives of the PACS		No. of items	Alpha^a^	Mean^b^	Range
**Quality**										
	Encouraging features	15	.906	3.284	1.567-4.033
	Non encouraging features	5	.767	3.000	2.50-3.400
**Information**										
	Produce better information	4	.888	3.754	3.650-4.000
**Images**										
	Quality of images produced	4	.910	4.272	4.183-4.333
	Compared to traditional films	4	.855	4.333	4.100-4.483
	Confidence in image quality	2	.875	4.205	4.154-4.256
	Data adequacy—access to patient data	2	.808	3.558	3.500-3.617
**Technical support**										
	Reliable, prompt services	7	.961	3.598	3.483-3.683
**Use of the PACS and satisfaction**										
	Frequency of PACS use	5	.638	3.573	2.583-4.000
	User satisfaction	3	.887	3.650	3.533-3.717
**Future use of the PACS**										
	Expectations, and continuance of use	3	.734	3.394	3.233-3.483
**Overall opinion and impact of the PACS**										
	Improved quality and services (benefits)	21	.919	4.008	3.169-4.390

^a^Cronbach Alpha: Measure of Internal Consistency Reliability.

^b^Mean values are based on a 5-point Likert scale, with 1 being the lowest and 5 being the highest.

#### Radiologists versus Technologists

Table 3 summarizes the comparison between radiologists’ and technologists’ responses with regard to their perspectives concerning the PACS. The mean values were significantly higher for the technologists as compared with the radiologists, especially concerning quality, information, patient data, technical support, and overall opinion on impact of the PACS (*P*<.05). Both professionals showed the highest level of satisfaction (mean >4) with regard to image produced, also their overall opinions on PACS demonstrated improved quality and services (radiologist 3.9 and technologists 4.2).

**Table 3:** Radiologists’ and technologists’ responses.

User perspectives on the PACS		Radiologists (n=39)		Technologists (n=21)		*P* value
		Mean^a^	SD	Mean^a^	SD	
**Quality**					
					
	Encouraging features	3.109	0.559	3.733	0.528	.006
	None encouraging features	3.070	0.693	3.333	0.563	.244
**Information**
					
	Produce better information	3.539	0.830	4.155	0.886	.007
**Images**
					
	Quality of images produced	4.188	0.692	4.429	0.598	.186
	Compared to traditional films	4.436	0.622	4.143	0.705	.083
	Confidence in image quality^b^	4.205	0.704	—	—	—
	Data adequacy—access to patient data	3.295	1.074	4.048	0.879	.005
**Technical support**					
					
	Reliable, prompt services	3.396	1.080	3.973	0.600	.029
**Use of the PACS and satisfaction**					
					
	Frequency of PACS use	3.585	0.760	3.552	0.819	.963
	User satisfaction	3.556	0.863	3.825	0.611	.144
**Future use of the PACS**					
					
	Expectations and continuance use	3.282	0.867	3.603	0.629	.140
**Overall opinion and impact of the PACS**					
					
	Improved quality and services (benefits)	3.892	0.623	4.218	0.427	.050

^a^Mean values are based on a 5-point Likert scale, with 1 being the lowest and 5 being the highest.

^b^Technologists were not asked this question, as the decision on image quality lies on radiologists.

**Figure 3:**
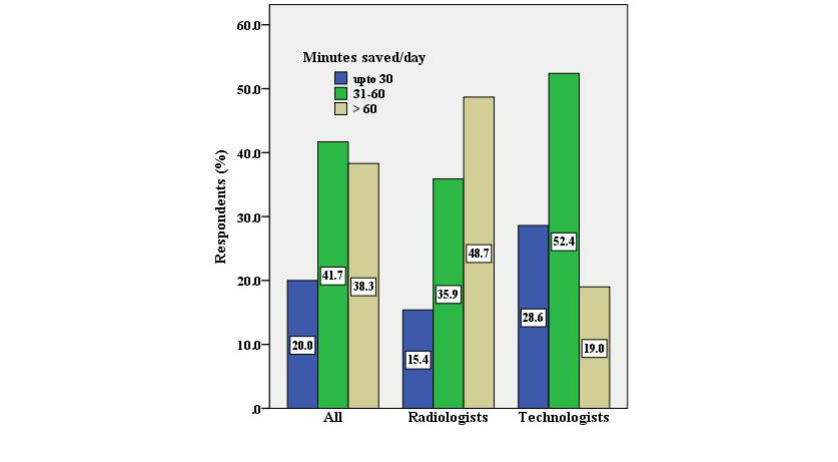
Respondents’ Minutes Saved per Day.

In total, 49% (19 of 39) of the radiologists mentioned saving more than 60 minutes every day, as compared to 19% (4 of 21) of the technologists (*P*=.048) (Figure 3).

**Figure 4:**
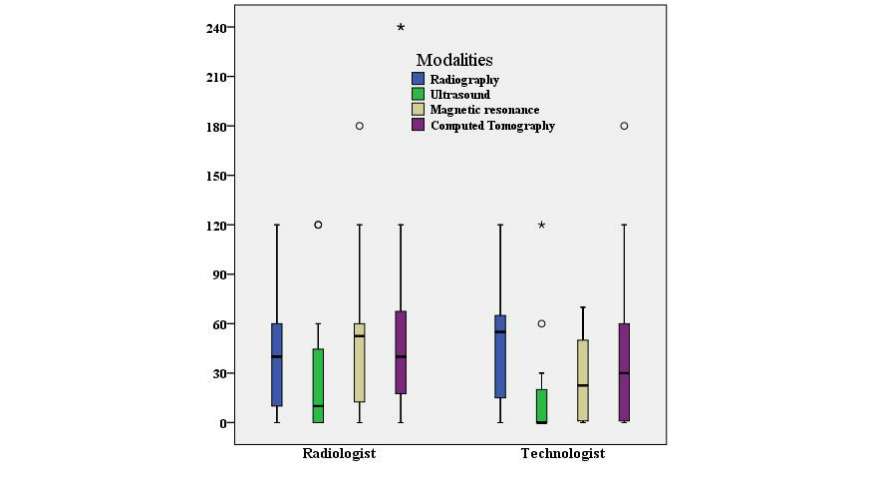
Average minutes (median with interquartile range) saved per day by picture archiving and communication system (PACS) users in different modalities.

During using the PACS, both the professionals reported a good saving in the working time for different modalities, though with much variation (the Kolmogorov–Smirnov tests showed a skewed distribution), the median and interquartile have been presented in Figure 4 as box-plot. The maximum number of minutes saved was 52 minutes (median time) by radiologists in magnetic resonance imaging and 50 minutes by technologists in radiography.

A significant positive correlation was observed between the number of hours using the PACS and the minutes saved in daily practice since the introduction of the PACS (*r*=0.27, *P*=.037).

The level of prior familiarity with computers was found to be similar between the radiologists (4.84±1.34 SD) and the technologists (4.71±1.35 SD) and did not make any significant difference either in the average duration (hours/week) of working with the PACS or the time saved (minutes/day) during practice.

The results of the open-ended questions showed that 24% (9 of 38) of the radiologists and 33% (7 of 21) of the technologists stated that storing, retrieving, and comparing images were the most positive elements associated with the use of the PACS. By contrast, 33% (13 of 39) of the radiologists and 43% (9 of 21) of the technologists stated that frequent glitches were the most negative element associated with the PACS.

Overall, the study’s findings revealed that both the radiologists and the technologists perceived the adoption of the PACS positively. The mean scores were mostly above 3 or 4 on a scale of 1-5. The mean scores for image quality and information produced were 4.3 and 3.8, respectively. The users seemed quite satisfied with the services and technical support, with a mean score of 3.6 and showed satisfaction in working with the PACS (mean=3.65). The PACS users clearly mentioned improved services and quality since the system came into practice, with a mean score of 4.

#### Interviews

The opinions of the PACS administrators were obtained by using the interview method, for which a series of semi structured questions on specific themes (Textbox 1) provided the basis for soliciting information.

At the time of the interviews, Mubarak Al-Kabeer Hospital had 5 PACS administrators: For scheduling the interviews, requisite permission was taken from the head of the radiology department, and interview sessions were arranged with the staff during their respective work breaks, over a 5-day period. Each interview session lasted approximately 50 minutes. The interviews were transcribed, and the responses were coded and analyzed using thematic analysis.

The interview results showed that all the interviewees had a BSc degree in radiological sciences, with their ages ranging between 25 and 35 years, and each having work experience of 2-5 years in PACS administration. Of the interviewees, only 3 had undertaken an introductory training program abroad on PACS use and management.

#### Perceived System Quality

The interview responses confirmed that the PACS provided easy access to authorized users, each with a user identification (ID) number and password, thereby providing a secured workspace depending on the user’s position. For instance, a radiology technologist’s access is limited to only viewing the reporting screen, with no authorization to change or manipulate it, thus preserving the data, with no hacking or security problems ever encountered or reported.

The interviewees unanimously agreed that the PACS was user friendly and hassle free in its functionality. In one of the interviewee’s words, “*We haven’* *t experienced any complaints from radiologists regarding the clarity of the PACS’s features, or any difficulties in moving between its functions*,” further adding that training in the PACS should be a prerequisite before its use.

**Figure 5:**
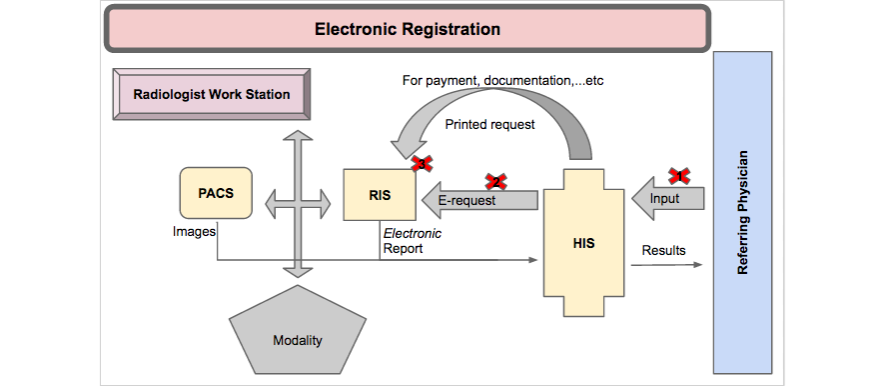
Workstations where electronic registrations of patients failed through the health information system (HIS) and radiology information system (RIS).

The participants also endorsed the reliability and consistency of the existing hardware, including computer systems, networks, and printers, with the software used. The interviews further revealed that the PACS was fully integrated and compatible with the RIS and the HIS, although the workflow did not follow the planned process, as Figure 5 demonstrates. According to one interviewee, “*The real mistakes are not coming from the PACS but from humans, so they’re human errors.*” The interview responses also highlighted that the problems associated with PACS integration and compatibility with the RIS and the HIS were the result of disorganized workflow, as shown in Figure 5.

**Figure 6:**
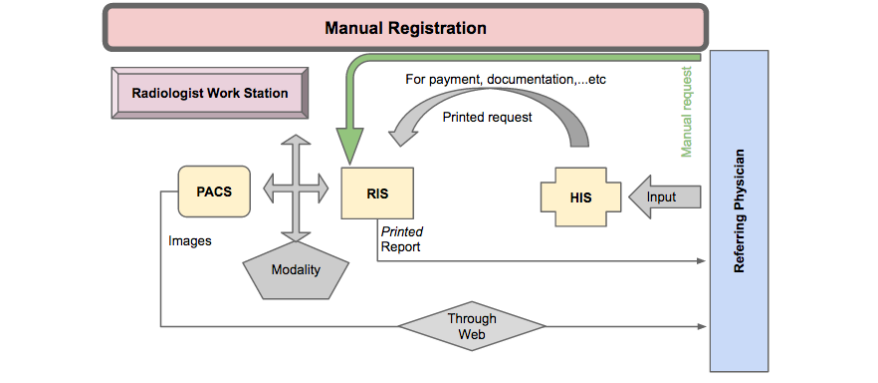
Manual registration of patients through the health information system (HIS) and radiology information system (RIS).

Figure 5 (above) illustrates the workstations where electronic registration of patients through the HIS, and the RIS failed due to receptionist errors such as: (1) no data entry into HIS, manual registration in the RIS; (2) failure of communication between the HIS and the RIS, manual registration in the RIS; (3) and incorrect registration at reception, manual registration in the RIS (Figure 6).

The manual registration at these 3 workstations resulted in: (1) a lack of direct access to patients’ imaging results through the HIS; (2) the creation of multiple PACS numbers for the same patient, making it difficult to retrieve previous reports for comparison, as well as the loss of patient data; and (3) delayed patient case management due to a failure in the rapid delivery of results.

#### Perceived Information Quality

The interviewees agreed that the PACS provided a standard format for the acquisition of accurate and complete information, together with images, concerning the patients’ medical cases, including their name, age, gender, national identification number, medical record number, and medical history. The lapses that occurred in the recorded information were attributed to the registration staff of the diagnostic radiology department because of their noncompliance to instructions, which resulted in incomplete data records of patients at the time of registration.

#### Perceived Image Quality

The interview responses indicated that one of the main roles of PACS administrators was to ensure that the images were transferred and displayed with clarity to facilitate studying and reporting. The participants further confirmed that “*We experience hangs in the images in PACS, but at an acceptable rate*” and no complaints were mentioned concerning image manipulation and management.

#### Perceived Technical Support Services

As the interviewees mentioned, the main IT support is delivered through the company that sold the PACS. This usually happens when the PACS administrators face a technical problem that can only be solved through the main IT support at the company. Thus, the PACS administrators asked to have some power to authorize them to solve the technical issues within the radiology department. One of the interviewees stated: “*…even when we want to connect a new printer to the PACS, we have to call the main IT support to perform this function for us.*” However, all of the interviewees complimented the IT support services at the company for their prompt responses to any technical issues.

#### Impact of PACS on Clinical Practice

PACS has an impact on the clinical practice of radiologists and technologists, as shown in the interviews' results

#### Perceived Net Benefits of the PACS

From the interviews, it was easy to see that the PACS has increased users’ productivity in comparison to the traditional filming system by minimizing their effort and time. In addition, the retaking of images is not required, as the PACS facilitates image storage and retrieval faster and over a longer period. “*We are happy with the PACS’* *s benefits*,” reported one interviewee, although the system has slowed in speed due to the huge number of cases, with the intervening procedures passing through several modalities, such as computed tomography and magnetic resonance imaging. There is also the possibility of missed images, especially concerning unknown IDs, although these could be traced using the patient’s civil ID, the patient’s PACS ID, or the excision ID of images.

#### User Satisfaction with the PACS

All the interviewees were apparently satisfied with the PACS; however, the technology-associated problems need to be addressed to optimize the system’s versatility and performance.

#### Opinions on the PACS

Overall, from the interviews, the responses revealed that as long as the image is electronically collected, stored, and communicated to another system successfully, the productively of work will be increased, diagnosis will be precisely performed, the patient will be treated accurately and quickly, and health services will be improved.

#### Expectations of the Current PACS and Future Trends

The interviewees expressed satisfaction in using the PACS system but also highlighted the need for resolving the current problems, as well as to keep abreast of the latest advances in PACS operations, to meet the growing demands of the Kuwaiti health system. The emerging requirements for potential trends in the future concern the areas of: (1) teleradiology services (for radiologists to use the PACS anywhere and anytime); (2) mobile images viewer for faster accessibility to images; (3) speech recognition functions; (4) computer-assisted diagnosis (CAD); (5) advanced training; and (6) recruiting health informatics graduates to support the PACS administrators.

## Discussion

In general, the study’s findings revealed that the PACS has had a productive impact on the staff’s clinical practice. Despite some of the technical limitations of the infrastructure, most of the respondents rated the system positively and as user friendly. The findings showed that the technologists were more satisfied than the radiologists were with using the PACS. Interestingly, there was a significant relationship between the perceived benefits of the PACS and the willingness of users to continue using it. It was also noteworthy that the problems associated with the PACS’s integration with the RIS were the result of disorganized workflow.

The results of the study revealed that the users’ demographic data, including computer experience, had no influence on their response patterns, being insignificant determinants of their predilection or preference for the PACS in enhancing their work efficiency. These findings were consistent with the study’s results on PACS acceptance [7], but contradicted with the results of earlier studies that reported the significant influence of age and gender on users’ choices concerning information technology, such as computer use patterns [20,21], particularly to adopting PACS [3,8].

### Perceptions of PACS Quality, Information, Images, and Services

The study further revealed that both the radiologists and the technologists were satisfied with the quality of information and images produced and had positive views regarding the use of this technology. The PACS offered the users with the requisite information on a medical case and facilitated the accomplishment of several functions with efficiency and ease in producing high-quality images with precision and clarity. This positive relationship found between users' satisfaction and quality of information and images, produced by PACS was consistent with the findings of previous studies [1,22]. The results of the interviews further complemented these findings, with no mention of lost images posing a major problem, due to successful image retrieval by PACS administrators.

The study found that the technologists were more satisfied than the radiologists, concerning their current PACS use, attributing their satisfaction to 2 reasons, which had been confirmed in previous studies [2,6,23]: the technologists achieved their core objectives of using the PACS, including image access, storage, and retrieval and (2) the radiologists looked beyond these features for additional facilities and functions, such as the PACS being packaged with CAD, teleradiology, or speech recognition functions. As the radiologists had been using the PACS far longer than the technologists had, their understanding and familiarity with the PACS appeared to be relatively higher.

Concerning the quality of the services offered to support PACS technically, the findings showed that both users were satisfied with the technical support provided with regard to the promptness, reliability, and dependability of the services. However, the results of the interviews revealed that the radiologists and the technologists encountered organizational and infrastructure deficiencies. On the technical level, there was frequent breakdown of the system during rush hours; and on organizational level, there was negligence of some receptionists in recording patients’ information from the RIS to the HIS. Interestingly, the respondents still showed satisfaction in confirming the benefits of the PACS over conventional radiology despite some deficiencies, as reportedly addressed in previous studies [3,22].

### Perceptions of the PACS’s Impact, Including Net Benefits and User Satisfaction

Regarding the PACS’s net benefits, the findings demonstrated that both the radiologists and the technologists had used the PACS to enhance their work productivity with ease due to the swift storage, retrieval, and transfer of images along with reports. These findings were consistent with those reported in previous evaluative studies on the impact of PACS [6,24], confirming that work productivity in regard to the given effort, time, and accuracy of reporting, has obviously been improved. Furthermore, the PACS’s benefits were found to have direct implications for user satisfaction, affecting their continued use of the PACS in the future [16]. These previous studies concluded that the more the users agreed with a PACS’s effectiveness in their work, the more they were satisfied and willing to continue using it. The findings of the interviews further confirmed that both types of users benefitted from the PACS’s advantages, expressing their readiness toward the technology’s continuous use while looking ahead for additional functions, without deficiencies, which coincided with other studies [25-27].

### Limitations

(1) This study was limited to radiologists and radiology technologists and did not involve other health care providers who are responsible for receiving patients’ reports and images. Hence, there is a need for further research that would substantiate the study’s findings by involving other stakeholders using the PACS facility, for the purpose of comparing research outcomes and enhancing the study’s value. (2) The study also did not include socioeconomic and cultural factors, which are significant predictors of IT adoption in the Arab world [7,28,29] in comparison to Western countries. However, the respondents’ willingness to use the PACS was a positive indicator of the technology’s versatility, efficiency, and continuous use. (3) As the study was confined to one general hospital in Kuwait, there is a definitive need for future studies to enhance the study’s scope by including other hospitals where PACSs are being used, for comparative purposes. (4) The study used specific criteria in evaluating IS success; hence, there is a need for using different models and tools for exploring and assessing PACSs and RISs from different dimensions.

### Conclusion

Evaluating the applications of imaging informatics, such as PACSs, in hospitals is very crucial to ensure the successful implementation of the applications, to identify the systems’ strengths and weaknesses during operation and to provide the opportunity for further improvements, strengthening the positive elements and minimizing drawbacks.

The evaluation of the existing PACS at Mubarak Al-Kabeer teaching hospital led to the successful assessment of the technology’s implications, based on which the study’s conclusions are summarized: (1) the PACS exhibited a positive impact on the radiologists and the technologists in the diagnostic radiology department, significantly enhancing their work efficiency and productivity. Therefore, the impact of the technology was particularly visible in the context of its ability to store and retrieve images quickly, enabling the users to accomplish their tasks swiftly. In addition, the system facilitated the addition of an image to a report, expediting communication with another location with a keystroke; (2) the main concern reported by all the users was the frequent breakdown during rush hours at busy workstations, due to infrastructure deficiency; (3) both the technologists and the radiologists indicated the need for a more-advanced PACS in response to the growing demand of teleradiology, mobile image viewer, and voice recognition features; and (4) evaluating PACS’s success is not confined to the technology itself but also concerns organizational and human factors that could limit the full integration with HIS.

### Recommendations

To improve the work on the current PACS and overcome the deficiencies, the following recommendations could be considered at Mubarak Al-Kabeer general hospital: (1) the need to enhance the capacity of existing servers to accommodate the huge amount of data generated from the massive inflow of patients. (2) The need to develop an internal policy to facilitate the coordination with the hospital management for organizing hospital workflow with efficiency. This policy should be followed carefully by the department staff for achieving the full benefits of the PACS’s integration with the HIS and the RIS. (3) The need to offer advanced training courses for fully using the PACS’s functions. (4) The need to look forward for future trends of PACS, including teleradiology services, mobile images viewer, speech recognition functions, and CAD. (5) The need to hire health informatics specialists for providing the requisite administrative support on account of their knowledge in the field.

## Abbreviations

CAD: computer-assisted diagnosis
HIS: health information system
ID: identification
IS: information system
PACS: picture archiving and communication System
RIS: radiology information system
